# Self-Other Differences in Perceiving Why People Eat What They Eat

**DOI:** 10.3389/fpsyg.2017.00209

**Published:** 2017-02-15

**Authors:** Gudrun Sproesser, Verena Klusmann, Harald T. Schupp, Britta Renner

**Affiliations:** Department of Psychology, University of KonstanzKonstanz, Germany

**Keywords:** eating motives, healthy eating, optimistic bias, peer-perception, self-perception, self-other bias, unrealistic optimism, self-favoring bias

## Abstract

People often view themselves more favorably than others, displaying unrealistic optimism. In the present study, we investigated whether people perceive their reasons for eating as better than those of others. Furthermore, we investigated which mechanisms of inaccuracy might underlie a possible bias when perceiving why people eat what they eat. In Study 1, 117 participants rated the social desirability of eating motives. In Study 2, 772 participants provided information on their own and others’ motives for eating behavior. In Study 1, particularly desirable motives were eating because of hunger, health reasons, and liking. Particularly undesirable motives were eating to make a good impression, to comply with social norms, and to regulate negative affect. Study 2 revealed that for socially desirable motives, participants perceived their own motives to be stronger; for undesirable motives, the opposite pattern emerged, with others being attributed stronger motives. Moreover, the perception of others’ emotional and social motives varied with participants’ own healthy eating behavior. Since the perception of eating motives of others should be independent of one’s own behavior, this pattern of results indicates a relative inaccuracy in the perception of others’ eating motives. In conclusion, there is evidence for unrealistic optimism in eating motives. For social and emotional motives, this self-favoring view seems to be driven by a relatively inaccurate perception of others.

## Introduction

Recently, The Telegraph published an article titled “Britain’s poor diet more deadly than its smoking habit …" (Donelly, 2015). Such statements are often based on research findings on unhealthy eating habits (e.g., Krebs-Smith et al., 2010). However, most people report their eating as being healthier than average (Sparks et al., 1995; Paisley and Sparks, 1998; Sproesser et al., 2015a). On the individual level, such a favorable view might be accurate. On the group level, however, it represents an unrealistic bias: If all people claim their eating is healthier than average, “they are clearly making a systematic error" (Weinstein, 1980, p. 806). This better-than-average-phenomenon has been demonstrated for perceptions of behaviors and behavioral consequences, such as health risks, and has been named ‘optimistic bias’ or ‘unrealistic optimism’ (for overviews see Miles and Scaife, 2003; Renner and Schupp, 2011; Shepperd et al., 2013). However, little is known on whether people are also ‘optimistic’ when perceiving behavioral precursors. For instance, do people also perceive the reasons why they eat what they eat more favorably than those of others? Specifically, people might believe that their own motives are better than those of others by perceiving that their food selection is, for example, more often based on health reasons than that of others.

Such a self-favoring perception of one’s own eating motives in comparison to others’ might be of concern because people might be less motivated to adopt a healthy eating style, as research on optimistic biases in risk perception suggests (e.g., Weinstein, 1982; Davidson and Prkachin, 1997; Shepherd, 2002; Dillard et al., 2009; Leikas et al., 2009). In a similar vein, Sparks et al. (1995) argued that an overestimation of one’s own as compared to one’s peers’ healthy eating might prevent diet modification (see also Oenema and Brug, 2003).

### How do People Perceive Their Own Compared to Others’ Reasons for Behavior?

Considerable social psychology research shows that perceptions of why people behave in a certain way are biased. For instance, Heider (1958) suggested that perceived reasons tend to “fit the wishes” of the perceiver (p. 172). According to the self-serving attributional bias, attributions for the self are biased, attributing success to our own dispositions and failure to external forces (Miller and Ross, 1975; see also Mezulis et al., 2004). Moreover, the attribution of others’ behavior has been shown to be biased. Specifically, people overestimate dispositions and underestimate situational influences as the reasons for others’ behavior choices, known as fundamental attribution error (Jones and Nisbett, 1972; Ross, 1977). Hence, the question arises whether people are also biased in perceiving the reasons for their own and the reasons for others’ behavior when it comes to eating.

Regarding reasons for eating behavior, there is a large variety of motives why people eat what they eat, such as health-related motives (Steptoe et al., 1995), economic reasons (French, 2003; Marquis, 2005), emotional motives (van Strien et al., 1986; Kröller et al., 2013; Meule et al., 2014; Keller and Siegrist, 2015), or social motives (Herman et al., 2003; Jackson et al., 2003; Robinson et al., 2011). The degree of social desirability might vary across these different motives: Some are probably seen positively, some are probably seen negatively, and others as neutral, having neither positive nor negative valence. For instance, eating for health reasons might constitute a desirable motive as eating healthily is generally recommended by national Nutrition Societies (e.g., DGE, 2016), health professionals, and the media. Similarly, attitudes regarding natural or ethical food choice motives have been found to be generally positive (Tarkiainen and Sundqvist, 2005). In contrast, economic motives might be undesirable, as they often conflict with desirable environmental and health concerns by preferring cheap and convenient foods (e.g., Carolan, 2011). In a similar vein, using food intake to create a certain impression typically might constitute an undesirable motive, as it has been discussed to lead to chronic food restriction and unhealthy eating habits (Vartanian et al., 2007). Relatedly, eating to comply with social norms contrasts with the general human motivation for self-determined behavior (Deci and Ryan, 2000) and might, thus, be a rather undesirable motive. Also, emotional eating is largely seen as a maladaptive eating motive that contributes to weight gain (e.g., Koenders and van Strien, 2011).

### Which Pattern Supports Unrealistic Optimism in the Reasons for Eating Behavior?

There are two possibilities regarding potential self-other differences in the perception of eating motives. First, people might, on average, perceive some of their own eating motives as stronger than those of others and some as weaker, regardless of a positive, negative, or neutral valence. Such valence-independent self-other differences could result from a lack of knowledge of others. This is one potential source of the fundamental attribution error (see the classical work of Jones and Nisbett, 1972; see also Gilbert and Malone, 1995). Second, people might, on average, perceive positive motives as being stronger for themselves than for others. Accordingly, they might perceive negative motives as being weaker for themselves than for others. Such systematic self-other differences as a function of motive desirability would clearly speak in favor of a bias and unrealistic optimism in eating motives (cf., Weinstein, 1980; Renner et al., 2015; Sproesser et al., 2015a,b).

### Which Mechanisms Might Underlie Unrealistic Optimism in Eating Motives?

Unrealistic optimism can stem from three different mechanisms. First, people might perceive themselves favorably and others accurately, indicating an optimistic self-bias (see, for example, Epley and Dunning, 2000; Balcetis and Dunning, 2013). Second, people might perceive themselves accurately and others unfavorably, indicating a pessimistic peer-bias (see, for example, Rothman et al., 1996; Lally et al., 2011; Sproesser et al., 2015b; Gamp and Renner, 2016). And third, people might perceive both themselves and others inaccurately (for an overview see Chambers and Windschitl, 2004). To test whether the self- or peer-perception is inaccurate, self- and others-views need to be assessed separately. Within this indirect method to assess unrealistic optimism, ratings for the self and the peer are compared on the group level (Perloff and Fetzer, 1986; Renner and Schupp, 2011; Shepperd et al., 2013).

With respect to eating motives, it is methodologically challenging to test whether absolute levels of self- and peer-perception are accurate. However, separate investigation of self- and peer-motives across different types of eaters allows testing relative accuracy in perceptions. For example, in risk research it was found that people who demonstrated a risky behavior (e.g., smoking) perceived themselves as more at risk than people with a healthier behavioral pattern. The observed covariation between one’s own behavior and risk perceptions for the self indicates relative accurate risk perceptions: people are aware of their risk status (see also Brewer et al., 2004; Renner et al., 2008)^1^. In a similar vein, compared to unhealthy eaters, people who reported a healthier eating behavior reported, on average, more pronounced health-related eating motives and less pronounced emotional eating motives (Pollard et al., 1998; Eertmans et al., 2005; Konttinen et al., 2010; Sproesser et al., 2011). Hence, people who report stronger health-related eating motivations actually also demonstrate a (self-reported) healthier eating pattern. This systematic variation between healthy eating behavior and specific self-related eating motives indicates a relative accuracy in perceived self-related eating motives across participants. However, a different rationale applies for the perceived motives for others, since from a normative perspective, peer-related perceptions should not vary with the behavioral status of the participant. Accordingly, a relative *in*accuracy in perceiving others’ motives is indicated if the perception varies with one’s own eating behavior. For example, when participants with an unhealthy diet ascribe others a less pronounced health motive than do those with a healthy diet, they demonstrate relative inaccuracy in the perception of others.

### The Present Studies

The aim of Study 1 was to investigate the perceived desirability of eating motives. We hypothesized that eating because of health or natural concerns are desirable eating motives, while eating to create a good impression or to comply with social norms, as well as eating to regulate negative emotions and economic motives (i.e., habits, convenience, price) are undesirable eating motives.

In Study 2, we aimed to investigate how people, on average, perceive others’ eating motives in comparison to their own eating motives. We hypothesized that people have a self-favoring view of their eating motives, perceiving desirable motives as stronger for the self than for others and conversely, perceiving undesirable motives as weaker for the self than for others. Moreover, we aimed to investigate the relative accuracy of self- and peer-views, that is, which motives vary with the eating behavior of the self.

## Study 1

### Methods

In Study 1, 117 psychology students (91 women) of the University of Konstanz were asked to rate the degree of desirability of eating motives. This sample had a mean age of 21.6 years (*SD* = 6.1, ranging from 18 to 63 years). The mean BMI was 21.4 kg/m^2^ (*SD* = 2.7, ranging from 17.1 to 37.0 kg/m^2^). Participants’ years of education ranged from 13 to 18 years with *M (SD)* = 13.9 (1.7), including schooling plus vocational or university training.

Eating motives were selected from The Eating Motivation Survey (TEMS, Renner et al., 2012b; Sproesser et al., submitted). TEMS comprises 78 (full version) and 45 (brief version) items designed to assess the 15 basic eating motives Health, Natural Concerns, Liking, Need and Hunger, Pleasure, Habits, Sociability, Weight Control, Convenience, Visual Appeal, Traditional Eating, Price, Affect Regulation, Social Norms, and Social Image. In order to keep the burden for participants as low as possible, one item was chosen for each of the 15 motive factors with regard to content validity and factor loading. Participants were instructed to judge the extent to which each of the 15 eating motives was desirable. A desirable motive was defined as a “good” reason why people eat what they eat, an undesirable motive as a “bad” reason (cf., Alicke, 1985 for the desirability of trait adjectives). For example, the desirability of the Health motive was assessed by the item ‘To eat what you eat because it is healthy is ….’ Responses were given on a seven-point rating scale from 1 ‘not at all desirable’ to 7 ‘very desirable.’

Statistical analyses were conducted using IBM SPSS statistics software (Version 23.0 for Windows). In order to test which motives are perceived as desirable and which are perceived as undesirable, single sample *t*-tests were computed, testing whether mean motive scores significantly deviated from the scale mean (i.e., a value of 4), which indicates that the motive is neither desirable nor undesirable. All tests were based on a 5% significance level.

### Results

Means, standard deviations, and test of perceived desirability of eating motives are displayed in **Table 1**. Motives that were perceived to be desirable were eating because of health reasons, natural concerns, liking, hunger, pleasure, sociability, and traditional eating. Motives that were perceived to be undesirable were weight control reasons, visual appeal, economic motives (i.e., habits, convenience, price), affect regulation, social norms, and social image. The three most desirable motives (in descending order) were to eat for reasons of need and hunger, health, and liking; the three least desirable motives were eating because of social image, social norms, and to regulate negative affect.

**Table 1:** Means, standard deviations, and test of desirability of eating motives.

Motive	*M*	*SD*	*t*(116)^a^	*p*	*d*
**Health-related motives**					
Health	6.03	1.22	17.95	<0.001	1.66
Natural Concerns	5.48	1.44	11.14	<0.001	1.03
Weight Control	3.53	1.34	-3.81	<0.001	0.35
**Sensory-biological motives**					
Liking	5.57	1.22	13.94	<0.001	1.29
Need and Hunger	6.41	0.91	28.36	<0.001	2.62
Visual Appeal	3.15	1.44	-6.37	<0.001	0.59
**Economic motives**					
Habits	3.72	1.37	-2.23	0.028	0.21
Convenience	3.55	1.35	-3.63	<0.001	0.34
Price	3.15	1.38	-6.71	<0.001	0.62
**Emotional motives**					
Pleasure	5.26	1.34	10.20	<0.001	0.94
Affect Regulation	2.30	1.38	-13.34	<0.001	1.23
**Social motives**					
Sociability	5.13	1.36	9.00	<0.001	0.83
Traditional Eating	4.94	1.28	7.93	<0.001	0.73
Social Norms	2.02	1.15	-18.62	<0.001	1.72
Social Image	1.83	1.05	-22.30	<0.001	2.06

^a^*Statistics refer to testing the deviation of motive means from scale means (i.e., a value of 4). Responses were given on a scale from 1 ‘not at all desirable’ to 7 ‘very desirable.’*

## Study 2

### Methods

#### Design and Procedure

Data were collected as part of the Konstanz Life-Study, a longitudinal cohort study launched in spring 2012 with 1,321 participants recruited in Konstanz, Germany (see also Renner et al., 2012a; Sproesser et al., 2015a; Klusmann et al., 2016; Sproesser et al., submitted). The Konstanz Life-Study was part of the projects EATMOTIVE and SmartAct funded by the Federal Ministry of Education and Research (BMBF Grants 01EA1326 and 01EL1420A, granted to BR and HS). At Wave 1, 1,321 participants were recruited via flyers, posters, and newspaper articles. Waves 2 and 3 took place in autumn 2012 and spring 2013. Participants were invited via email or phone to re-attend. The three measurement points included the collection of questionnaires, blood samples, and a standardized checkup including anthropometric measures and functional and cognitive fitness tests. This study presents data on participants’ own eating motives and those perceived for an average person as well as self-reported eating behavior at Wave 2^2^.

#### Sample

At Wave 2, 772 participants (58% female, *n* = 447) provided data on their eating motives and those of an average person. This sample had a mean age of 47.7 years (*SD* = 17.4, ranging from 19 to 87 years). The mean BMI was 24.8 kg/m^2^ (*SD* = 3.9, ranging from 17.3 to 45.8 kg/m^2^). Participants’ years of education ranged from 8 to 20 years with *M (SD)* = 15.8 (2.4), including schooling plus vocational or university training, assessed at Wave 1. Compared with the German population (Statistisches Bundesamt, 2016a,b), this sample was 4 years older, had 7% more females, and had a lower average BMI (average BMI of the German population = 26 kg/m^2^ according to 2009 Microcensus data).

The study sample did not differ from the drop-out sample at Wave 1 (*N* = 549) regarding gender (58% vs. 61% women, χ^2^(1) = 0.88, *p* = 0.350) or BMI (25.0 vs. 24.6 kg/m^2^, *t*(1305) = 1.72, *p* = 0.085). On average, the study sample was 8 years older than the drop-out sample (47.1 vs. 39.0 years, *t*(1311) = 8.42, *p* < 0.001) and slightly better educated (15.8 vs. 15.3 years of education, *t*(1284) = 3.19, *p* = 0.001).

All participants gave written informed consent prior to data collection and the ethics board of the University of Konstanz approved the study protocol. For data processing and security, a register of processing operations was developed in cooperation with and approved by the Center for Data Protection of the Universities in Baden-Württemberg (ZENDAS, Zentrale Datenschutzstelle der baden-württembergischen Universitäten) and reviewed by the Commissioner for Data Protection in Baden-Württemberg (Landesdatenschutz Beauftragte, Baden-Württemberg) in 2012. The procedures were performed in compliance with relevant laws and institutional guidelines. We followed the German Psychological Society’s (Deutsche Gesellschaft für Psychologie) guidelines for conducting psychological studies^3^ (see paragraph C.III). These correspond to those of the American Psychological Association. The study conforms to the Declaration of Helsinki.

#### Measures

##### Motives of the self and the peer

Motives of the self and an average person of the same age and gender (the peer) were assessed with TEMS (Renner et al., 2012b; Sproesser et al., submitted). The measurement of self- and peer-ratings was conducted in accordance with the indirect method of assessing an optimistic bias (Perloff and Fetzer, 1986). In line with Study 1, one item was selected for each of the 15 motives to assess the peer’s motives. The brief version of TEMS (45 items) was used to assess people’s own motives. For the current manuscript, we compared the corresponding 15 items for the self to the 15 items for the peer. For example, for the Health motive we compared the items ‘I eat what I eat because it is healthy’ (motive of the self) and ‘An average person of my age and gender eats what he/she eats because it is healthy’ (motive of the peer). Responses were given on a seven-point rating scale from 1 ‘never’ to 7 ‘always.’

##### Eating behavior

Eating behavior was assessed with a validated food frequency questionnaire (Winkler and Döring, 1995, 1998; see also Sproesser et al., 2011, 2015a). Participants were asked how often on average they eat food items from 15 selected categories (e.g., wholemeal products, vegetables, fruits, chocolate, cake, meat, and salty snacks), ranging from 1 ‘nearly once a day’ to 6 ‘never.’ The consumption frequency of these 15 food categories was categorized as optimal, regular, and unfavorable according to recommendations from the German Nutritional Society (Winkler and Döring, 1995). This categorization was accumulated into a food frequency index reflecting dietary quality with a possible range of 0–30. The index was classified into an optimal (scores of 16 or higher; *n* = 373), regular (scores greater than 13 and lower than 16; *n* = 177), and unfavorable (scores of 13 or below; *n* = 222) dietary pattern. This classification has been validated using 7-day dietary records from the WHO MONICA Augsburg Dietary Survey (see Winkler et al., 1991; Winkler and Döring, 1995).

#### Analytical Procedure

Statistical analyses were conducted using IBM SPSS statistics software (Version 23.0 for Windows). Participants were excluded from data analyses if they filled in less than 75% of the questionnaire (*n* = 27). Missing data in questionnaire variables were imputed using the Expectation Maximization algorithm in SPSS (Gold and Bentler, 2000). Missing data in demographics were not imputed. Missing values were below 5% for all imputed variables. In order to test the research questions, analyses of variance (ANOVAs) including simple main effects were computed. In case of violation of the sphericity assumption, Greenhouse-Geisser correction was applied. All tests were based on a 5% significance level. All analyses were secured with gender, age, and BMI as control variables. As results remained unchanged, analyses without these control variables are reported for brevity.

### Results

#### Perception of Others’ and Own Eating Motives

Means and standard deviations for participants’ perceptions why others and why they themselves eat what they eat are displayed in **Table 2**. On average, participants reported that they often to very often eat what they eat because of sensory-biological motives such as taste (Liking) or hunger (Need and Hunger) as well as because of health reasons (Health), with mean values above 5 (see **Table 2**). The emotional motive to regulate negative affect (Affect Regulation) as well as social motives such as complying with social norms (Social Norms) or making a good impression (Social Image) were rated as very rarely impacting own eating behavior, with mean values below 3 (**Table 2**). This pattern is similar to results from other samples using the full EMS (e.g., Renner et al., 2012b; Sproesser et al., submitted).

**Table 2:** Means, standard deviations, and differences between the motives for the self and the peer.

	Self		Peer					
Motive	*M*	*SD*	*M*	*SD*		*F*(1,771)^a^	*p*	$\eta_{p}^{2}$
**Health-related motives**					
Health	5.11	1.19	4.39	1.09		195.98	<0.001	0.20
Natural Concerns	4.47	1.73	3.93	1.21		64.79	<0.001	0.08
Weight Control	3.28	1.53	4.28	1.16		205.89	<0.001	0.21
**Sensory-biological motives**					
Liking	5.48	0.96	5.20	0.85		47.74	<0.001	0.06
Need and Hunger	5.27	1.14	5.24	0.98		0.22	0.636	-
Visual Appeal	3.31	1.38	4.65	1.19		520.85	<0.001	0.40
**Economic motives**					
Habits	4.54	1.40	5.22	0.83		181.77	<0.001	0.19
Convenience	3.94	1.42	5.10	1.03		393.22	<0.001	0.34
Price	3.32	1.45	4.85	1.12		605.68	<0.001	0.44
**Emotional motives**					
Pleasure	4.45	1.25	4.97	1.00		114.42	<0.001	0.13
Affect Regulation	2.43	1.37	4.06	1.12		794.83	<0.001	0.51
**Social motives**					
Sociability	4.00	1.44	4.83	1.01		221.82	<0.001	0.22
Traditional Eating	3.05	1.36	4.49	1.22		707.18	<0.001	0.48
Social Norms	1.93	1.12	3.74	1.21		1108.46	<0.001	0.59
Social Image	1.90	1.01	3.78	1.28		1253.38	<0.001	0.62

^a^*Statistics refer to simple main effect analyses of target (self vs. peer) in the 15 × 2 ANOVA with the within-subject factors motives and target. Responses were given on a scale from 1 ‘never’ to 7 ‘always.’*

Regarding the perception of others’ motives, participants perceived that their peers often eat what they eat because of the sensory-biological motives taste (Liking) and hunger (Need and Hunger) as well as because of economic motives such as habits (Habits) or convenience (Convenience), with mean values above 5 (**Table 2**). Interestingly, there were no mean values below 3, indicating that participants, on average, did not rate any motive as rarely impacting their peers’ eating behavior (**Table 2**). The motives rated as impacting peers’ eating behavior least often, Social Norms and Social Image, were still rated with mean values of approximately 4, meaning that they sometimes impact peers’ eating behavior (see **Table 2**). Consequently, the means of the 15 motives had a considerably larger range for the self (1.90 – 5.48) than for the peer (3.74 – 5.24). Moreover, there were two large differences in the ranking orders: health was ranked third for the self and tenth for the peer; natural concerns fifth for the self and thirteenth for the peer.

#### Do People Have a Self-Favoring View of Their Eating Motives?

To test whether participants perceived their own desirable motives (e.g., eating because of health or natural concerns) as stronger than those of others and their own undesirable motives (e.g., eating to create a good impression or to comply with social norms) as weaker than those of others, a 15 × 2 ANOVA with the within-subject factors motives and target (self vs. peer) was calculated. There were significant main effects for motives, *F*(10.74, 8278.58) = 653.18, *p* < 0.001, $\eta_{p}^{2}$ = 0.46, and target, *F*(1,771) = 1228.63, *p* < 0.001, $\eta_{p}^{2}$ = 0.61. The main effect of motives indicated that some motives were rated as impacting eating behavior more often than others, independent of whether they were rated for the self or the peer. The main effect of target showed that peers’ motives were rated as being, on average, more pronounced than motives for the self. However, these main effects were qualified by a significant motive × target interaction, *F*(10.09, 7777.39) = 268.57, *p* < 0.001, $\eta_{p}^{2}$ = 0.26.

**FIGURE 1:**
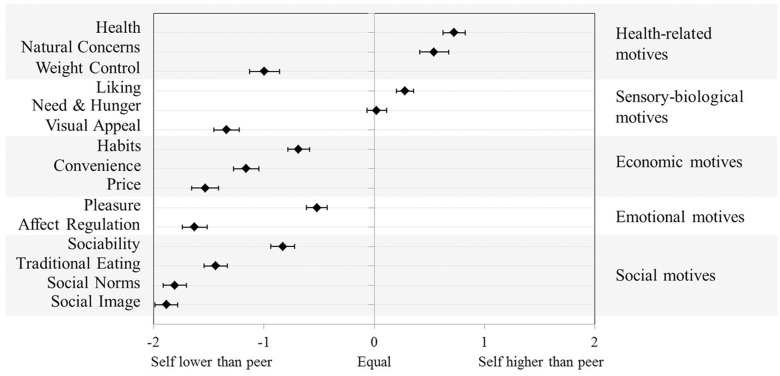
**Difference scores of motives for the self and the peer**. Positive vs. negative values indicate a stronger vs. weaker motive for the self than for the peer. Error bars indicate 95% confidence intervals.

To probe the interaction and clarify which motives differ between the self and the peer, simple main effects for the factor target (within the factor motives) were subsequently calculated (see **Table 2**). Furthermore, difference scores were calculated for self- and peer-motives to detail the results: positive values indicate a stronger self- than peer-motive, a zero value indicates that both motives were rated equally, and negative values indicate a weaker self- than peer-motive (see **Figure 1**).

Regarding health-related motives, participants believed that their food choices are based on health and natural concerns significantly more often than those of an average peer (see **Table 2** and **Figure 1**). Participants rated weight control reasons as lower for their own eating than for their peers’ eating. With respect to sensory-biological motives, participants reported that they are motived by taste significantly more often than their peers, whereas a reversed pattern was observed regarding visual appeal as a motive for eating. There was no self-other difference in the perceptions of eating due to hunger. Furthermore, participants believed that economic reasons, namely habit, convenience, and price, drive their eating significantly less often than their peers’ eating. The same held for emotional (i.e., eating for pleasure and to regulate negative affect) and social reasons (i.e., eating because of sociability, traditions, social norms, or social image concerns). Taken together, the desirable motives for eating Health, Natural Concerns, and Liking were rated stronger for the self than for the peer, whereas the undesirable motives (Weight Control, Visual Appeal, Habits, Convenience, Price, Affect Regulation, Social Norms, and Social Image) were rated weaker for the self than for the peer. This pattern speaks in favor of unrealistic optimism in the perceived reasons why people eat what they eat. Interestingly, for the desirable motive Need and Hunger, the own motive was not rated stronger than the peer’s motive. Also, the desirable motives Pleasure, Sociability, and Traditional Eating were rated weaker for the self than for the peer.

#### Relative Accuracy of Self- and Peer-Views: Variation with Healthy Eating Behavior

In a next step, we investigated relative accuracy in participants’ self- and peer-views, that is, which motives vary with healthy eating behavior. Therefore, the ANOVA with the within-subject factors motives and target (self vs. peer) was extended by the between-subjects factor dietary pattern (unfavorable, regular, optimal). This 15 × 2 × 3 ANOVA again revealed significant main effects of motives, *F*(10.82, 8318.95) = 593.92, *p* < 0.001, $\eta_{p}^{2}$ = 0.44, and target, *F*(1,769) = 1112.69, *p* < 0.001, $\eta_{p}^{2}$ = 0.59, as well as a significant motives × target interaction, *F*(10.40, 7997.85) = 235.32, *p* < 0.001, $\eta_{p}^{2}$ = 0.23. Moreover, there was a significant motives × dietary pattern interaction, *F*(21.64, 8318.95) = 4.23, *p* < 0.001, $\eta_{p}^{2}$ = 0.01, as well as a significant three-way interaction, *F*(20.80, 7997.85) = 7.59, *p* < 0.001, $\eta_{p}^{2}$ = 0.02. Neither the main effect of dietary pattern nor the target × dietary pattern interaction were significant, *F*(2,769) = 1.44, *p* = 0.237 and *F*(2,769) = 0.61, *p* = 0.545, respectively.

Probing the significant three-way interaction, simple main effect analyses of the factor dietary pattern within the motives for the self and the peer revealed four significant differences regarding self-motives and four significant differences for peers’ motives (see **Table 3**).

**Table 3:** Differences in motives for the self and the peer between participants with optimal, regular, and unfavorable dietary patterns.

	Self			Peer		
Motive	*F* (2,69)	*p*	$\eta_{p}^{2}$	*F*(2,69)	*p*	$\eta_{p}^{2}$
Health	39.33	0.000	0.09	0.30	0.741	
Natural Concerns	16.00	0.000	0.04	0.18	0.838	
Weight Control	16.91	0.000	0.04	0.31	0.731	
Convenience	10.73	0.000	0.03	0.06	0.939	
Pleasure	0.50	0.604		3.53	0.030	0.01
Affect Regulation	1.96	0.142		4.58	0.011	0.01
Traditional Eating	0.14	0.869		5.37	0.005	0.01
Social Image	0.51	0.600		3.10	0.046	0.01

*Statistics refer to simple main effects of dietary patterns within motives for the self and the average peer*.

**FIGURE 2:**
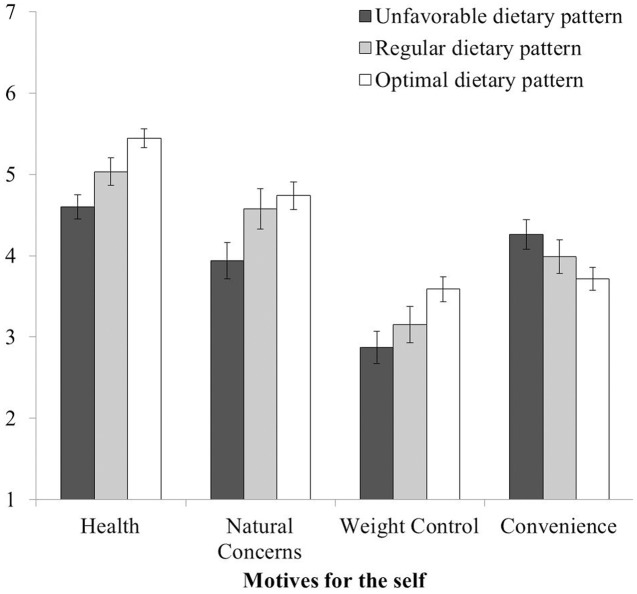
**Motives for the self that significantly varied with dietary pattern**. The response scale ranged from 1 (never) to 7 (always). Error bars indicate 95% confidence intervals.

With regard to motives for the self, participants with an optimal dietary pattern perceived their health-related motives (eating because of health, natural concerns, and weight control) as being stronger than did those participants with an unfavorable dietary pattern (see **Figure 2**). Among economic motives, choosing foods for convenience was stronger in people with an unfavorable dietary pattern than in those with an optimal dietary pattern. These results demonstrate relative accuracy in the self-view for these motives.

**FIGURE 3:**
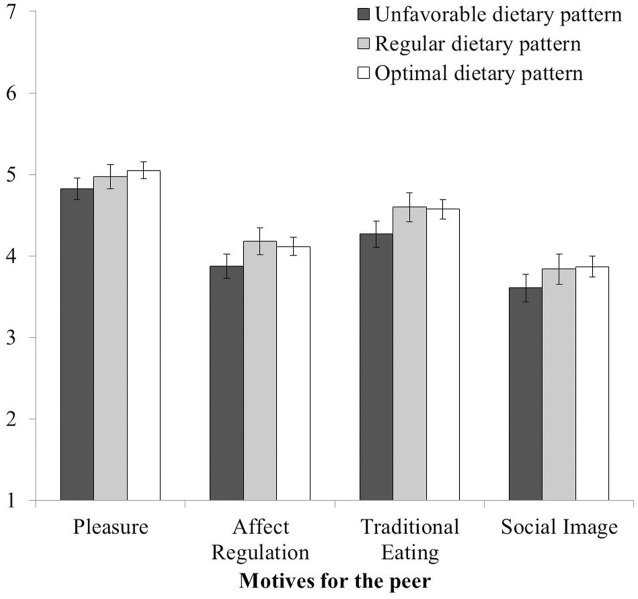
**Motives for the peer that significantly varied with dietary pattern**. The response scale ranged from 1 (never) to 7 (always). Error bars indicate 95% confidence intervals.

Regarding motives for the peer, people with an optimal dietary pattern perceived their peers as eating for pleasure or to regulate negative affect more often than did people with an unfavorable dietary pattern (see **Figure 3**). Similarly, people with an optimal dietary pattern rated peers’ social motives for choosing foods due to tradition or to make a good impression as stronger than did people with an unfavorable dietary pattern. These results indicate relative inaccuracy in the peer-perception of these motives. The remaining simple main effects of dietary pattern were not significant (*F*s ≤ 2.93; *p*s ≥ 0.054).

## Discussion

The present study investigated unrealistic optimism in the precursors of eating behavior, that is, why people eat what they eat, as well as its underlying mechanisms. In the case of desirable motives, such as eating for health reasons, participants perceived their own motives to be stronger than others’ motives. For undesirable motives, such as eating to regulate negative affect, the opposite pattern emerged, with stronger motives for others than for the self. This pattern speaks in favor of unrealistic optimism in the perception of why people eat what they eat. With regard to the underlying mechanisms, results indicated relative accuracy in the perception of one’s own health-related and convenience motives, but relative inaccuracy in the perception of others’ emotional and social motives. Relative accuracy was demonstrated by the finding that participants with optimal dietary pattern perceived their own eating behavior to be more often driven by health-related reasons and less often by convenience reasons than participants with an unfavorable dietary pattern. Relative inaccuracy occurred as healthy eaters also perceived others’ eating to be more often driven by emotional, traditional, and impression management reasons than did unhealthy eaters. Hence, there is evidence for unrealistic optimism in eating motives. For social and emotional motives, this self-favoring view seems to be driven by a relatively inaccurate perception of others.

### Optimism in Eating Motives

The presented results complement previous research on unrealistic optimism in behavior (e.g., Sparks et al., 1995; Paisley and Sparks, 1998; Sproesser et al., 2015a,b) and behavioral consequences (e.g., Miles and Scaife, 2003; Renner and Schupp, 2011; Shepperd et al., 2013). The observed systematic self-other differences in motives as a function of motive desirability speak in favor of an underlying systematic bias (cf., Weinstein, 1980; Renner et al., 2015; Sproesser et al., 2015a,b) rather than a lack of knowledge of others. Interestingly, the self-favoring view was most pronounced for the food choice motivations impression management, compliance with social norms, and regulating negative affect (see **Figure 1**). In contrast, it was least pronounced for hunger, taste, and habits. An explanation for this finding might be the following: According to the observed mean values of these motives, eating to make a certain impression, to comply with social norms, and to regulate negative affect occurs less frequently than eating because of hunger, taste, and habits. Weinstein (1980) and Rose et al. (2008) showed that the more frequently a hazard is perceived, the smaller the unrealistic optimism (see also Renner and Schupp, 2011). Hence, the differently pronounced self-favoring view as a function of the frequency with which motives drive behavior appears to be in line with previous research on determinants of unrealistic optimism.

Both motivational and cognitive processes have been purported to underlie unrealistic optimism (see Shepperd et al., 2002; Chambers and Windschitl, 2004; Renner and Schupp, 2011; Renner et al., 2015 for overviews). Motivational accounts postulate that a self-favoring bias is fueled by the motivation either to self-enhance or protect, and to maintain a positive view of oneself (e.g., Shepperd et al., 2002). An example for a cognitive process is the ‘egocentric bias.’ Hereby, a self-favoring bias might result from our tendency to focus more on our own characteristics than on those of others (Chambers and Windschitl, 2004).

All eight undesirable motives were rated stronger for the peer than for the self and three desirable motives were rated weaker for the peer than for the self. However, the desirable motive to eat because of hunger was not rated weaker for the peer than for the self. A reason for this finding might be that eating because of hunger is the prototypical motive why people eat what they eat and a biological necessity. Hence, it might be clear to people that everybody often eats because of hunger, not only they themselves but also other people. Interestingly, the desirable motives to eat because of pleasure, sociability, and traditions were even rated stronger for the peer than for the self. Motives to eat for pleasure, because it is social, or traditional have been shown to be more pronounced in younger and female participants (Renner et al., 2012b). Thus, these motives might be more pronounced in the sample of Study 1 than in the sample of Study 2. As higher values of a certain characteristic are likely to be related to a higher perceived desirability of this characteristic, eating for pleasure or because it is social or traditional might be perceived as desirable in the sample of Study 1, but not in the sample of Study 2. Thus, these counterintuitive ratings in terms of desirability and self-other differences might be due to assessing motive desirability and self-other differences in different samples.

### Stereotypes about Others Underlying a Biased Peer-Perception

An interesting finding was that the range of least to most frequent motives driving eating behavior was much smaller for others’ than for own motives. This result is probably driven by the fact that people have more information about themselves than they have about others. Moreover, it speaks in favor of the application of stereotypic schemata about others. Specifically, Kelley (1972) proposed that schemata are activated when it comes to the question why people behave in a certain way. A similar explanation might account for the observed relative inaccuracy in peers’ motives for eating because of pleasure, negative emotions, traditions, and social image concerns. More precisely, compared to unhealthy eaters, healthy eaters might more strongly envisage a stereotypical person with regard to these eating motives. For instance, healthy eaters might hold a clear stereotype of a person who eats when frustrated. As the activation of a stereotypical person has been associated with holding more extreme beliefs about a person (Weinstein, 1980; Perloff and Fetzer, 1986; Renner and Schupp, 2011; Renner et al., 2015), a stereotypical peer could be perceived as eating more often out of frustration than without this clear stereotype in mind. Taken together, the activation of stereotypes about others and schemata may account for a biased peer-perception underlying self-favoring eating motives (cf., Rothman et al., 1996; Lally et al., 2011; Sproesser et al., 2015b; Gamp and Renner, 2016). Future research is needed to figure out why peer-perception differed between healthy and unhealthy eaters for some motives but not others.

### Relative Accuracy of People’s Self-View

The systematic variation of the motives for the self to eat for health, natural, weight control, and convenience reasons with healthy eating behavior indicates relative accuracy in people’s self-view as this variation is in line with previous research (Pollard et al., 1998; Eertmans et al., 2005; Sproesser et al., 2011). However, people who reported healthy eating behavior did not differ from people displaying unhealthy eating behavior by habitually eating because of negative emotions or price concerns whereas an association between these two motives and healthy eating has been shown before (Pollard et al., 1998; Konttinen et al., 2010; Sproesser et al., 2011). This might indicate that people are also biased in the self-perception of these motives. For instance, a self-favoring view regarding the motive to regulate negative affect through eating might stem both from perceiving others unfavorably and oneself favorably. The latter self-bias might be indicated, as, contrary to evidence from previous research (e.g., Konttinen et al., 2010), unhealthy eaters did not report more emotional eating than healthy eaters. Another explanation might be that detrimental effects of habitually eating because of negative emotions or economic concerns may have been buffered by protective variables. For instance, emotional eating is but one factor contributing to food choice and dietary healthiness. A high tendency to regulate negative affect by eating can, however, be counteracted by high self-control (Sproesser et al., 2011). Hence, people with high emotional eating can nevertheless eat healthily if control resources are high. Whether one of these two or another explanation holds true needs to be clarified in future research.

### Limitations

Some limitations of this research need to be taken into account. Specifically, it is unknown whether our sample was representative in terms of eating motives. Thus, the discrepancy between the own and others’ average motives might reflect real group differences between our sample and the average population. However, the similarity of mean motives for the self in this study and previous research (e.g., Renner et al., 2012b) speaks against such a selective sampling effect. Furthermore, healthy eating was defined according to the recommendations of the German Nutritional Society (Winkler and Döring, 1995). However, at present, there is no commonly accepted definition of healthy or unhealthy eating, and thus, the classification is debatable. Moreover, the current study focused on motives for why people eat what they eat, but not on motives for the amount eaten. Additionally, the cross-sectional nature of the presented data does not allow for causal inferences regarding the relationship of optimism in eating motives and healthy eating behavior. Hence, future studies are needed to further elucidate perceptual biases in eating motives.

## Conclusion

The present research revealed that people believe that their own eating behavior is motivated by better reasons than that of others. Hence, when asked about the attributions of others’ behavior, people not only overestimate dispositions and underestimate situational influences (Jones and Nisbett, 1972; Ross, 1977; see also Funder, 2015) but also perceive others’ reasons less favorably than their own. Consequently, if the headline that people too rarely take health reasons into account when choosing foods was published, most people would probably still believe that they take health reasons into account more often than others. This could preclude the effectiveness of such warning messages and could explain a lack of reactions. Thus, future research should investigate the consequences of this optimism in why people eat what they eat.

## Author Contributions

VK, GS, and BR developed the study concept. All authors made substantial contributions to data acquisition. GS conducted data analyses and all authors contributed significantly to interpreting the data. GS prepared the first manuscript draft and all other authors provided critical revisions. All authors approved the final version of the manuscript for submission and agreed to be accountable for all aspects of the work.

## Conflict of Interest Statement

The authors declare that the research was conducted in the absence of any commercial or financial relationships that could be construed as a potential conflict of interest.

## Footnotes

1 It is important to note that perceptions might still be biased in an absolute sense – although people with a higher risk status might see themselves as being at greater risk, they still might underestimate their actual risk.

2 We did not include data from Waves 1 and 3 of the Konstanz Life-Study as the perception of others’ eating motives was only assessed at Wave 2.

3 http://www.dgps.de/index.php?id=96422

## References

[B1] Alicke M. D. (1985). Global self-evaluation as determined by the desirability and controllability of trait adjectives. *J. Pers. Soc. Psychol.* 49 1621–1630. 10.1037/0022-3514.49.6.1621

[B2] Balcetis E. Dunning D. (2013). Considering the situation: why people are better social psychologists than self-psychologists. *Self Identity* 12 1–15. 10.1080/15298868.2011.617886

[B3] Brewer N. T. Weinstein N. D. Cuite C. L. Herrington J. (2004). Risk perceptions and their relation to risk behavior. *Ann. Behav. Med.* 27 125–130. 10.1207/s15324796abm2702_7 15026296

[B4] Carolan M. (2011). *The Real Cost of Cheap Food.* Oxon: Routledge.

[B5] Chambers J. R. Windschitl P. D. (2004). Biases in social comparative judgments: the role of nonmotivated factors in above-average and comparative-optimism effects. *Psychol. Bull.* 130 813–838. 10.1037/0033-2909.130.5.813 15367082

[B6] Davidson K. Prkachin K. (1997). Optimism and unrealistic optimism have an interacting impact on health-promoting behavior and knowledge changes. *Pers. Soc. Psychol. Bull.* 23 617–625. 10.1177/0146167297236005

[B7] Deci E. L. Ryan R. M. (2000). The “What ” and “ Why ” of goal pursuits: human needs the self-determination of behavior. *Psychol. Inq.* 11 227–268. 10.1207/S15327965PLI1104_01

[B8] DGE (2016). *Vollwertig Essen und Trinken Nach den 10 Regeln der DGE.* Available at: https://www.dge.de/ernaehrungspraxis/vollwertige-ernaehrung/10-regeln-der-dge [accessed July 1 2016].

[B9] Dillard A. J. Midboe A. M. Klein W. M. P. (2009). The dark side of optimism: unrealistic optimism about problems with alcohol predicts subsequent negative event experiences. *Pers. Soc. Psychol. Bull.* 35 1540–1550. 10.1177/0146167209343124 19721102

[B10] Donelly L. (2015). *Britain’s Poor Diet More Deadly Than its Smoking Habit as Alcohol Related Deaths Soar.* Available at: http://www.telegraph.co.uk/news/health/news/11865074/Britains-poor-diet-more-deadly-than-its-smoking-habit-as-alcohol-related-deaths-soar.html [accessed on July 1 2016].

[B11] Eertmans A. Victoir A. Vansant G. Van den Bergh O. (2005). Food-related personality traits, food choice motives and food intake: mediator and moderator relationships. *Food Qual. Prefer.* 16 714–726. 10.1016/j.foodqual.2005.04.007

[B12] Epley N. Dunning D. (2000). Feeling “holier than thou”: Are self-serving assessments produced by errors in self- or social prediction? *J. Pers. Soc. Psychol.* 79 861–875. 10.1037/0022-3514.79.6.861 11138757

[B13] French S. A. (2003). Pricing effects on food choices. *J. Nutr.* 133 841–843. 10.1093/jn/133.3.841S 12612165

[B14] Funder D. C. (2015). Towards a de-biased social psychology: the effects of ideological perspective go beyond politics. *Behav. Brain Sci.* 38:e143 10.1017/s0140525x14001204 26786293

[B15] Gamp M. Renner B. (2016). Pre-feedback risk expectancies and reception of low-risk health feedback: absolute and comparative lack of reassurance. *Appl. Psychol. Health Well Being* 8 364–385. 10.1111/aphw.12076 27412477

[B16] Gilbert D. T. Malone P. S. (1995). The correspondence bias. *Psychol. Bull.* 117 21–38. 10.1037/0033-2909.117.1.21 7870861

[B17] Gold M. S. Bentler P. M. (2000). Treatments of missing data: a Monte Carlo comparison of RBHDI, iterative stochastic regression imputation, and expectation-maximization. *Struct. Equ. Model.* 7 319–355.

[B18] Heider F. (1958). *The Psychology of Interpersonal Relations.* New York, NY: John Wiley & Sons, Inc.

[B19] Herman C. P. Roth D. A. Polivy J. (2003). Effects of the presence of others on food intake: a normative interpretation. *Psychol. Bull.* 129 873–886. 10.1037/0033-2909.129.6.873 14599286

[B20] Jackson B. Cooper M. L. Mintz L. Albino A. (2003). Motivations to eat: scale development and validation. *J. Res. Pers.* 37 297–318. 10.1016/S0092-6566(02)00574-3

[B21] Jones E. E. Nisbett R. E. (1972). *The Actor and the Observer: Divergent Perceptions of the Causes of Behavior.* New York, NY: General Learning Press, 10.1037//0022-3514.71.2.375

[B22] Keller C. Siegrist M. (2015). Ambivalence toward palatable food and emotional eating predict weight fluctuations. Results of a longitudinal study with four waves. *Appetite* 85 138–145. 10.1016/j.appet.2014.11.024 25464025

[B23] Kelley H. K. (1972). “Causal schemata and the attribution process,” in *Attribution: Perceiving the Causes of Behavior*, eds Jones E. E. Kanhouse D. E. Kelley H. H. Nisbett R. E. Valins S. Weiner B. (Morristown, NJ: General Learning Press), 151–174.

[B24] Klusmann V. Musculus L. Sproesser G. Renner B. (2016). Fulfilled emotional outcome expectancies enable successful adoption and maintenance of physical activity. *Front. Psychol.* 6:1990 10.3389/fpsyg.2015.01990 PMC4701923 26779095

[B25] Koenders P. G. van Strien T. (2011). Emotional eating, rather than lifestyle behavior, drives weight gain in a prospective study in 1562 employees. *J. Occup. Environ. Med.* 53 1287–1293. 10.1097/JOM.0b013e31823078a2 22027541

[B26] Konttinen H. Männistö S. Sarlio-Lähteenkorva S. Silventoinen K. Haukkala A. (2010). Emotional eating, depressive symptoms and self-reported food consumption. A population-based study. *Appetite* 54 473–479. 10.1016/j.appet.2010.01.014 20138944

[B27] Krebs-Smith S. M. Guenther P. M. Subar A. F. Kirkpatrick S. I. Dodd K. W. (2010). Americans do not meet federal dietary recommendations. *J. Nutr.* 140 1832–1838. 10.3945/jn.110.124826 20702750 PMC2937576

[B28] Kröller K. Jahnke D. Warschburger P. (2013). Are maternal weight, eating and feeding practices associated with emotional eating in childhood? *Appetite* 65 25–30. 10.1016/j.appet.2012.11.032 23380038

[B29] Lally P. Bartle N. Wardle J. (2011). Social norms and diet in adolescents. *Appetite* 57 623–627. 10.1016/j.appet.2011.07.015 21843568

[B30] Leikas S. Lindeman M. Roininen K. Lähteenmäki L. (2009). Who is responsible for food risks? The influence of risk type and risk characteristics. *Appetite* 53 123–126. 10.1016/j.appet.2009.05.003 19433122

[B31] Marquis M. (2005). Exploring convenience orientation as a food motivation for college students living in residence halls. *Int. J. Consum. Stud.* 29 55–63. 10.1111/j.1470-6431.2005.00375.x

[B32] Meule A. Allison K. C. Platte P. (2014). Emotional eating moderates the relationship of night eating with binge eating and body mass. *Eur. Eat. Disord. Rev.* 22 147–151. 10.1002/erv.2272 24293184

[B33] Mezulis A. H. Abramson L. Y. Hyde J. S. Hankin B. L. (2004). Is there a universal positivity bias in attributions? A meta-analytic review of individual, developmental, and cultural differences in the self-serving attributional bias. *Psychol. Bull.* 130 711–747. 10.1037/0033-2909.130.5.711 15367078

[B34] Miles S. Scaife V. (2003). Optimistic bias and food. *Nutr. Res. Rev.* 16 3–19. 10.1079/NRR200249 19079933

[B35] Miller D. T. Ross M. (1975). Self-serving biases in the attribution of causality: Fact or fiction? *Psychol. Bull.* 82 213–225. 10.1037/h0076486

[B36] Oenema A. Brug J. (2003). Exploring the occurrence and nature of comparison of one’s own perceived dietary fat intake to that of self-selected others. *Appetite* 41 259–264. 10.1016/s0195-6663(03)00103-x 14637324

[B37] Paisley C. M. Sparks P. (1998). Expectations of reducing fat intake: the role of perceived need within the theory of planned behaviour. *Psychol. Health* 13 341–353. 10.1080/08870449808406755

[B38] Perloff L. S. Fetzer B. K. (1986). Self-other judgments and perceived vulnerability to victimization. *J. Pers. Soc. Psychol.* 50 502–510. 10.1037/0022-3514.50.3.502

[B39] Pollard T. M. Steptoe A. Wardle J. (1998). Motives underlying healthy eating: using the Food Choice Questionnaire to explain variation in dietary intake. *J. Biosoc. Sci.* 30 165–179. 10.1017/S0021932098001655 9746823

[B40] Renner B. Gamp M. Schmälzle R. Schupp H. T. (2015). “Health risk perception,” in *The International Encyclopedia of Social and Behavioral Sciences*, 2nd Edn, ed. Wright J. D. (Oxford: Elsevier).

[B41] Renner B. Schupp H. (2011). “The perception of health risks,” in *Oxford Handbook of Health Psychology*, ed. Friedman H. S. (New York, NY: Oxford University Press), 637–665.

[B42] Renner B. Schüz B. Sniehotta F. F. (2008). Preventive health behavior and adaptive accuracy of risk perceptions. *Risk Anal.* 28 741–748. 10.1111/j.1539-6924.2008.01047.x 18643829

[B43] Renner B. Sproesser G. Klusmann V. Schupp H. (2012a). Die Konstanzer Life-Studie. *Adipositas* 6 123–124.

[B44] Renner B. Sproesser G. Strohbach S. Schupp H. T. (2012b). Why we eat what we eat. The Eating Motivation Survey (TEMS). *Appetite* 59 117–128. 10.1016/j.appet.2012.04.004 22521515

[B45] Robinson E. Tobias T. Shaw L. Freeman E. Higgs S. (2011). Social matching of food intake and the need for social acceptance. *Appetite* 56 747–752. 10.1016/j.appet.2011.03.001 21396972

[B46] Rose J. P. Endo Y. Windschitl P. D. Suls J. (2008). Cultural differences in unrealistic optimism and pessimism: the role of egocentrism and direct versus indirect comparison measures. *Pers. Soc. Psychol. Bull.* 34 1236–1248. 10.1177/0146167208319764 18587057

[B47] Ross L. (1977). “The intuitive psychologist and his shortcomings: distortions in the attribution process,” in *Advances in Experimental Social Psychology* Vol. 10 ed. Berkowitz L. (New York, NY: Academic Press), 10.1016/S0065-2601(08)60357-3

[B48] Rothman A. J. Klein W. M. Weinstein N. D. (1996). Absolute and relative biases in estimations of personal risk. *J. Appl. Soc. Psychol.* 26 1213–1236. 10.1111/j.1559-1816.1996.tb01778.x

[B49] Shepherd R. (2002). Resistance to changes in diet. *Proc. Nutr. Soc.* 61 267–272. 10.1079/pns2002147 12133209

[B50] Shepperd J. A. Carroll P. Grace J. Terry M. (2002). Exploring the causes of comparative optimism. *Psychol. Belg.* 42 65–98.

[B51] Shepperd J. A. Klein W. M. P. Waters E. A. Weinstein N. D. (2013). Taking stock of unrealistic optimism. *Pers. Psychol. Sci.* 8 395–411. 10.1177/1745691613485247 PMC4451212 26045714

[B52] Sparks P. Shepherd R. Wieringa N. Zimmermanns N. (1995). Perceived behavioural control, unrealistic optimism and dietary change: an exploratory study. *Appetite* 24 243–255. 10.1016/S0195-6663(95)99787-3 7574571

[B53] Sproesser G. Klusmann V. Schupp H. T. Renner B. (2015a). Comparative optimism about healthy eating. *Appetite* 90 212–218. 10.1016/j.appet.2015.03.008 25770914

[B54] Sproesser G. Kohlbrenner V. Schupp H. Renner B. (2015b). I eat healthier than you: differences in healthy and unhealthy food choices for oneself and for others. *Nutrients* 7 4638–4660. 10.3390/nu7064638 26066013 PMC4488806

[B55] Sproesser G. Strohbach S. Schupp H. Renner B. (2011). Candy or apple? How self-control resources and motives impact dietary healthiness in women. *Appetite* 56 784–787. 10.1016/j.appet.2011.01.028 21296115

[B56] Statistisches Bundesamt (2016a). *Zahlen und Fakten.* Available at: https://www.destatis.de/DE/ZahlenFakten/ZahlenFakten.html [accessed July 1 2016].

[B57] Statistisches Bundesamt (2016b). *Informationssystem der Gesundheitsberichterstattung des Bundes.* Available at: http://www.gbe-bund.de [accessed July 1 2016].

[B58] Steptoe A. Pollard T. M. Wardle J. (1995). Development of a measure of the motives underlying the selection of food: the Food Choice Questionnaire. *Appetite* 25 267–284. 10.1006/appe.1995.0061 8746966

[B59] Tarkiainen A. Sundqvist S. (2005). Subjective norms, attitudes and intentions of Finnish consumers in buying organic food. *Br. Food J.* 107 808–822. 10.1108/00070700510629760

[B60] van Strien T. Frijters J. E. Bergers G. Defares P. B. (1986). The Dutch Eating Behavior Questionnaire (DEBQ) for assessment of restrained, emotional, and external eating behavior. *Int. J. Eat. Disord.* 5 295–315.

[B61] Vartanian L. R. Herman C. P. Polivy J. (2007). Consumption stereotypes and impression management: how you are what you eat. *Appetite* 48 265–277. 10.1016/j.appet.2006.10.008 17157957

[B62] Weinstein N. D. (1980). Unrealistic optimism about future life events. *J. Pers. Soc. Psychol.* 39 806–820. 10.1037/0022-3514.39.5.806

[B63] Weinstein N. D. (1982). Unrealistic optimism about susceptibility to health problems. *J. Behav. Med.* 5 441–460. 10.1007/BF00845372 7154065

[B64] Winkler G. Döring A. (1995). Kurzmethoden zur Charakterisierung des Ernährungsmusters: Einsatz und Auswertung eines Food-Frequency-Fragebogens. *Ernährungsumschau* 42 289–291.

[B65] Winkler G. Döring A. (1998). Validation of a short qualitative food frequency list used in several German large scale surveys. *Z. Ernährungswiss.* 37 234–241. 10.1007/s003940050022 9800314

[B66] Winkler G. Döring A. Keil U. (1991). Differences in dietary intake between weekend and weekdays - results from the MONICA-project Augsburg Dietary Survey 1984/85. *Z. Ernährungswiss.* 30 313–317. 1788999 10.1007/BF01651961

